# Cognitive trajectory patterns in oldest‐old individuals for identifying SuperAgers: The Life After 90 Study

**DOI:** 10.1002/alz.093353

**Published:** 2025-01-03

**Authors:** Roshni Biswas, Luohua Jiang, Claudia H. Kawas, Paola Gilsanz, Rachel A. Whitmer, María M. M. Corrada

**Affiliations:** ^1^ University of California Irvine, Irvine, CA USA; ^2^ University of California, Irvine, Irvine, CA USA; ^3^ Kaiser Permanente Northern California Division of Research, Oakland, CA USA; ^4^ University of California, Davis, Davis, CA USA

## Abstract

**Background:**

Oldest‐old individuals exhibit heterogeneous cognitive function ranging from superior cognitive performance to dementia. Most SuperAger definitions rely on cross‐sectional memory scores. Here, we extend the definition by identifying distinct subgroups based on longitudinal trajectories for memory. We also evaluated longitudinal trajectories for executive function.

**Method:**

The 774 study participants were from the LifeAfter90 study (LA90), a multi‐ethnic cohort of individuals aged 90 or older. Verbal episodic memory (VEM) and executive function (EF) were assessed approximately every 6 months using the Spanish and English Neuropsychological Assessment Scale from at least two visits (mean = 4; range: 2‐7). Using group‐based trajectory modelling, we identified unique trajectories separately for VEM and EF, accounting for baseline age, sex, education, and race/ethnicity.

**Result:**

62% of participants were women, the overall mean baseline age was 92.4 (±2.3), and 26% had ≤high school education (Table 1). We identified four distinct trajectories for VEM (Figure 1). Group 4‐High stable performers: with the highest baseline performance, largest practice effects, and no decline; Group 3‐Stable performers: with a lower baseline than group 4, moderate practice effects, and slight decline; Group 2‐Slow decliners: with lower baseline than groups 4 and 3, slight practice effects, and a slow declining trajectory; and Group 1‐Accelerated decliners: with the lowest baseline performance, no practice effects, and rapid decline. Thus, we identified one subgroup with no decline in longitudinal performance and with practice effects that could be classified as SuperAgers. For EF, we also identified four groups (Figure 2). Baseline EF scores varied for Groups 4, 2, and 1 (Group 4 being the highest) and followed relatively stable trajectories. Group 3, which had the second highest baseline score, had a slowly declining trajectory. For EF, stable trajectories were not limited to the top baseline performing groups. The two domains had distinct patterns with limited overlap in group memberships.

**Conclusion:**

Using longitudinal patterns, we identified SuperAgers based on superior memory performance and no decline. In future research, we will further explore the use of longitudinal performance trajectories and the use of multiple cognitive domains in the definition of SuperAgers. We will also identify defining characteristics of each subgroup.

 [Table alz093353-tbl-0001], [Fig alz093353-fig-0001], [Fig alz093353-fig-0002]

**TABLE 1 alz093353-tbl-0001:** Baseline characteristics of study participants.

Characteristics	Total (n = 774)
Sex	
Male	291 (38%)
Female	483 (62%)
Age at baseline	
Mean (SD)	92.4 (2.3)
Education	
Less than or equal High school	205 (26%)
Trade school/some college	294 (38%)
College or more	275 (86%)
Race	
African American	174 (22%)
Asian	184 (24%)
Caucasian	225 (29%)
Latino	139 (18%)
Multiple/Others	55 (7%)
Total number of visits	
Mean (SD)	4.0 (1.8)

**FIGURE 1 alz093353-fig-0001:**
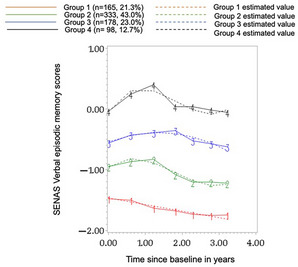
Four latent trajectories for Verbal episodic memory in Life After 90 study participants identified by Group‐based trajectory modeling. Age at baseline, sex, education, and race were included in the model to account for their influence in group membership.

**FIGURE 2 alz093353-fig-0002:**
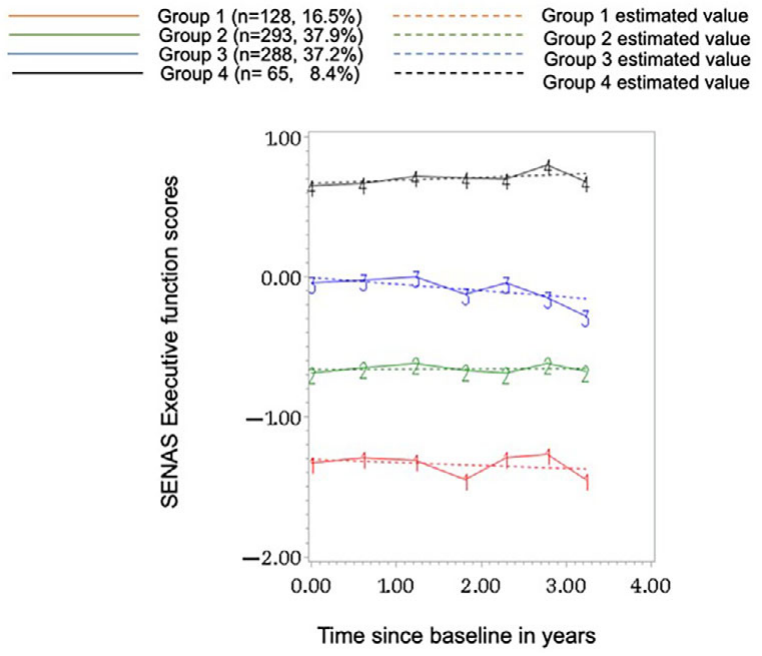
Four latent trajectories for Executive function in Life After 90 study participants identified by Group‐based trajectory modeling. Age at baseline, sex, education, and race were included in the model to account for their influence in group membership.

